# Bupivacaine reduces GlyT1 expression by potentiating the p-AMPKα/BDNF signalling pathway in spinal astrocytes of rats

**DOI:** 10.1038/s41598-022-05478-3

**Published:** 2022-01-26

**Authors:** Kaimei Lu, Liyan Zhao, Yonghai Zhang, Fan Yang, Huiwen Zhang, Jie Wang, Bin Li, Guimei Ji, Jianqiang Yu, Hanxiang Ma

**Affiliations:** 1 Department of Anesthesiology, Ningxia Medical University, Yinchuan, 750004 China; 2 Department of Anesthesiology, People’s Hospital of Ningxia Hui Autonomous Region, Yinchuan, 750004 China; 3 Department of Anesthesiology, General Hospital of Ningxia Medical University, Yinchuan, 750004 China; 4 Department of Pharmacology, College of Pharmacy, Ningxia Medical University, Yinchuan, 750004 China

**Keywords:** Neuroscience, Medical research, Molecular medicine

## Abstract

Bupivacaine, a local anaesthetic, is widely applied in the epidural or subarachnoid space to clinically manage acute and chronic pain. However, the underlying mechanisms are complex and unclear. Glycine transporter 1 (GlyT1) in the spinal cord plays a critical role in various pathologic pain conditions. Therefore, we sought to determine whether bupivacaine exerts its analgesic effect by regulating GlyT1 expression and to determine the underlying mechanisms of regulation. Primary astrocytes prepared from the spinal cord of rats were treated with bupivacaine. The protein levels of GlyT1, brain-derived neurotrophic factor (BDNF) and phosphorylated adenosine 5′-monophosphate (AMP)-activated protein kinase α (p-AMPKα) were measured by western blotting or immunofluorescence. In addition, 7,8-dihydroxyflavone (7,8-DHF, BDNF receptor agonist) and AMPK shRNA were applied to verify the relationship between the regulation of GlyT1 by bupivacaine and the p-AMPKα/BDNF signalling pathway. After treatment with bupivacaine, GlyT1 expression was diminished in a concentration-dependent manner, while the expression of BDNF and p-AMPK was increased. Moreover, 7,8-DHF decreased GlyT1 expression, and AMPK knockdown suppressed the upregulation of BDNF expression by bupivacaine. Finally, we concluded that bupivacaine reduced GlyT1 expression in spinal astrocytes by activating the p-AMPKα/BDNF signalling pathway. These results provide a new mechanism for the analgesic effect of intrathecal bupivacaine in the treatment of acute and chronic pain.

## Introduction

Intrathecal bupivacaine is considered a treatment option in acute and chronic pain management^1–3^. However, the underlying mechanisms are more complex than simply blocking voltage-gated Na^+^ channels. Bupivacaine, in addition to blocking sodium channels, also affects the activity of many other membrane proteins and channels^4–6^. Moreover, previous studies have demonstrated that local anaesthetic can diminish glycine uptake mediated by glycine transporter 1 (GlyT1)^7,8^, which plays an important role in neuropathic pain. However, it is unclear whether the analgesic mechanism of intrathecal bupivacaine is related to the regulation of GlyT1 expression.

Glycine is a major inhibitory neurotransmitter in the central nervous system (CNS) and controls motor and sensory signal transduction. The glycine concentration in the synaptic cleft is strictly controlled by glycine transporters (GlyTs), including GlyT1 and GlyT2. GlyT1 is mainly expressed in astrocytes but also reported in neurons, whereas GlyT2 is exclusively expressed in glycinergic neurons^9^. At glycinergic synapses, GlyT1 plays a role in the removal of glycine from the synaptic cleft, while GlyT2 transports glycine from the synaptic cleft back into glycinergic neuron terminals. In addition, GlyT1 mediates excitatory glutamatergic neurotransmission by removing glycine from the glutamatergic synaptic cleft^10^.

The important role of glycine inhibitory neurotransmission in central pain control has been demonstrated^11^. Increasing evidence suggests that the activity of inhibitory neurons in the spinal cord is compromised in neuropathic and inflammatory pain states^12^, and restoring this inhibition can reverse pain sensitivity. Likewise, the facilitation of inhibitory glycinergic neurotransmission through GlyT inhibition has become a potential novel treatment of acute and chronic pain^13–16^. In particular, inhibition of GlyT1 not only promotes glycine concentration in the glycine synaptic cleft but also reduces the expression of N-methyl-D-aspartate receptors (NMDARs) in the glutamate synapse to ameliorate neuropathic pain^17^.

GlyT1 expression is modulated by various factors, one of which is that BDNF promotes GlyT1 endocytosis and degradation and inhibits GlyT1-mediated glycine uptake^18^. BDNF is one of the major neurotrophic factors in the development, maturation, and maintenance of the CNS. Moreover, it modulates the morphology of astrocytes by interacting with tropomyosin-receptor-kinase B (TrkB)^19,20^. Furthermore, BDNF expression is controlled by p-AMPKα in the CNS^21,22^. AMPK is an important intracellular energy sensor involved in energy metabolism. The specific phosphorylation of AMPK can activate transcriptional regulators to affect protein expression. Furthermore, local anaesthetics have been demonstrated to activate AMPK^23–25^.

Therefore, we speculated that bupivacaine might decrease GlyT1 expression by activating the p-AMPKα/BDNF signalling pathway. To verify this hypothesis, we used primary astrocytes from the rat spinal cord to study the effect of bupivacaine on GlyT1, and the expression levels of GlyT1, BDNF, and p-AMPKα were detected in astrocytes after treatment with bupivacaine. In addition, we used 7,8-DHF or AMPK shRNA to modulate related protein expression. Finally, the results supported our hypothesis that bupivacaine decreased GlyT1 expression in astrocytes, which was associated with the activation of the p-AMPKα/BDNF signalling pathway. Thus, modulation of GlyT1 expression might represent a novel mechanism for the antinociceptive action of intrathecal bupivacaine.

## Results

### Bupivacaine regulated GlyT1 and BDNF expression in primary astrocytes

**Figure 1:**
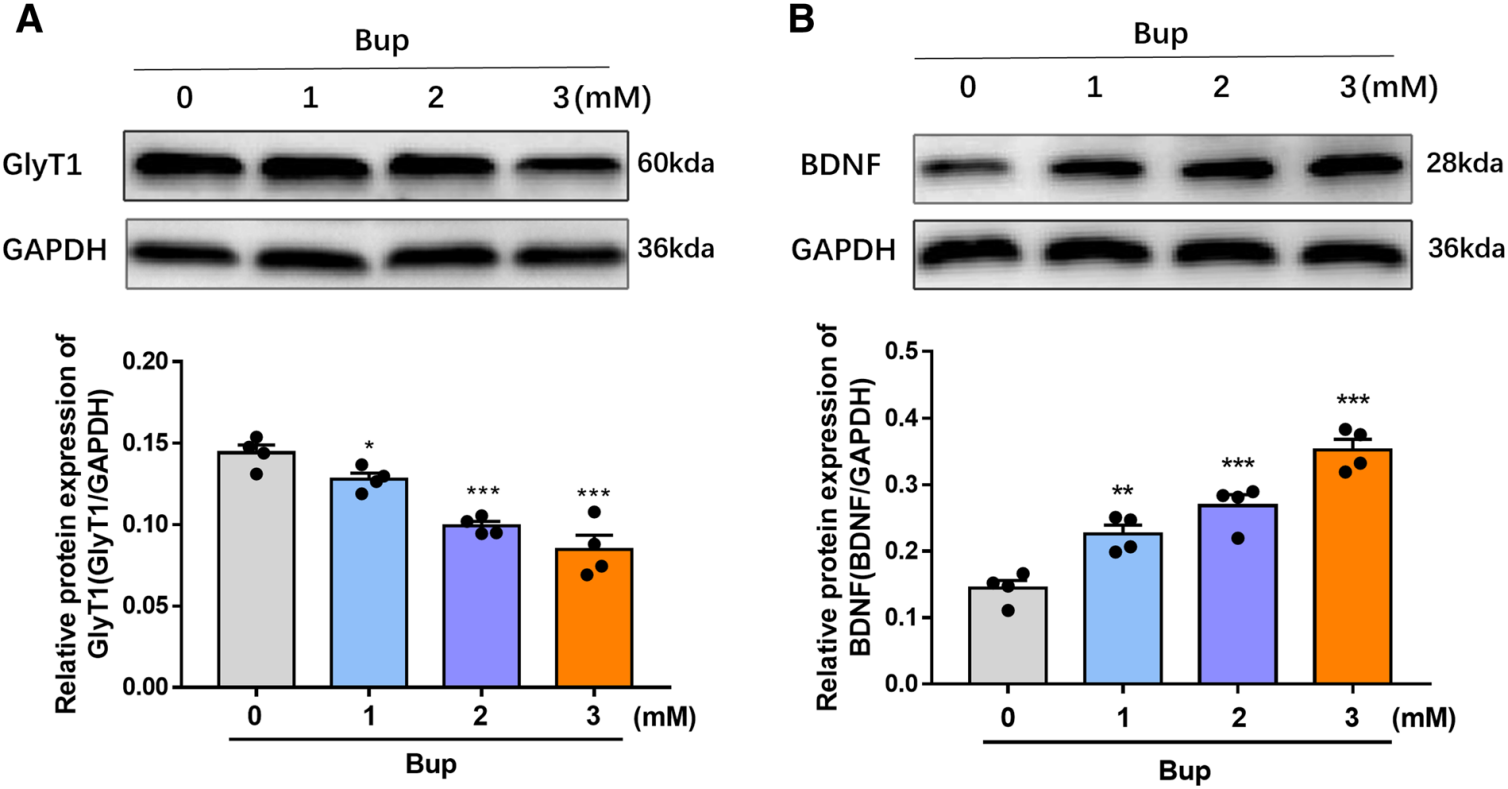
Bupivacaine regulated GlyT1 and BDNF expression in astrocytes. (**A**,**B**) Representative western blotting and quantification of GlyT1 and BDNF expression. *p < 0.05, **p < 0.01, ***p < 0.001 vs. the control group. Data are expressed as the mean ± SEM. Original images of gels/blots are available in Supplementary Fig S1. Bup, Bupivacaine.

The regulation of GlyT1 and BDNF expression by bupivacaine was determined in primary astrocytes derived from the spinal cord of rats. Data from western blot analysis indicated a prominent decrease in GlyT1 expression at different concentrations of bupivacaine for 2 h (*p < 0.05, ***p < 0.001, Fig. 1A). In addition, the most significant decrease in GlyT1 expression was in the 3 mM bupivacaine group. The expression of BDNF was increased significantly after treatment with bupivacaine, especially in the 3 mM bupivacaine group (*p < 0.05, **p < 0.01, ***p < 0.001, Figs. 1B and 3B).

### Treatment with 7,8-DHF decreased GlyT1 expression in astrocytes

**Figure 2:**
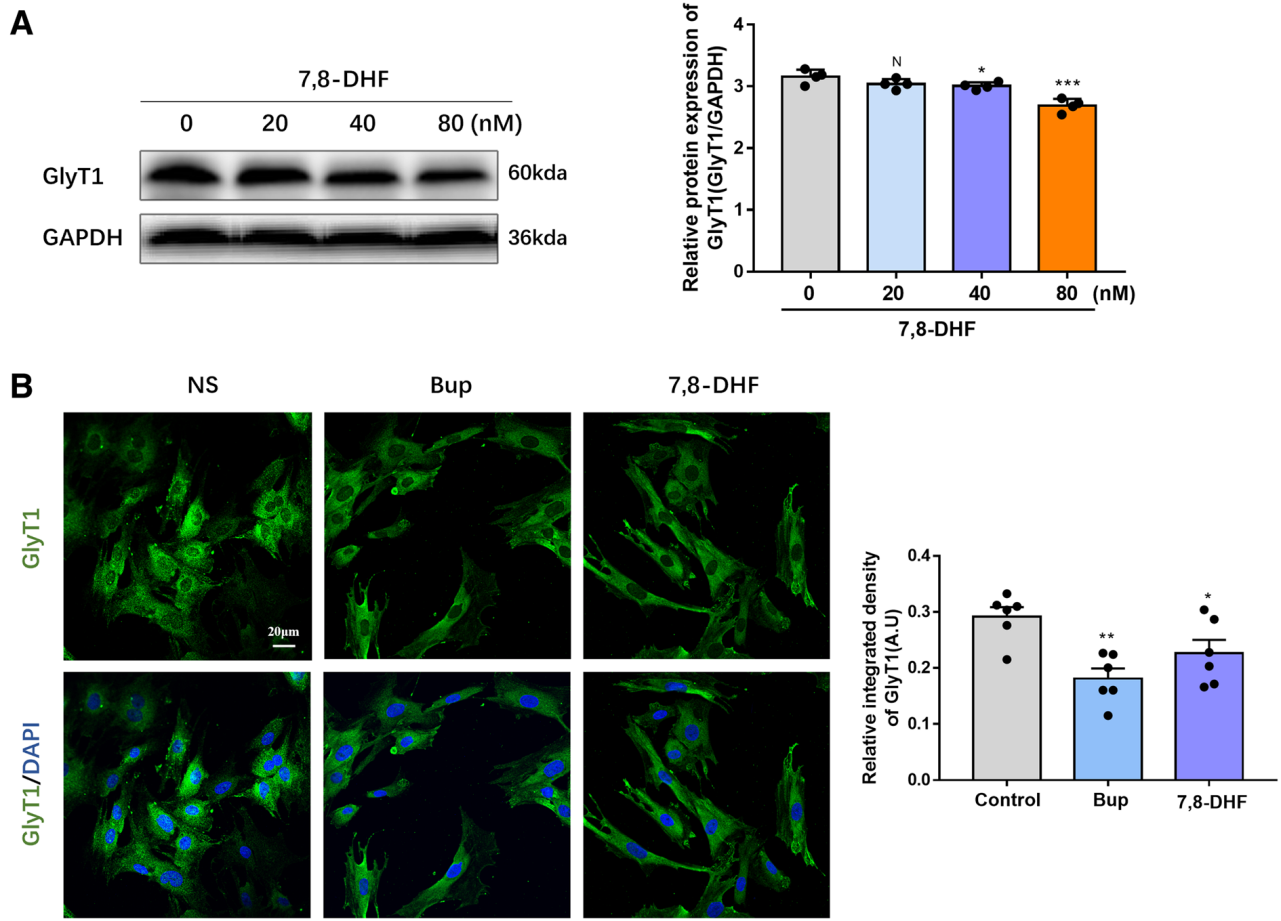
Effects of 7,8-DHF (BDNF receptor agonist) on GlyT1 in astrocytes. (**A**) Protein expression of GlyT1 in astrocytes after treatment with 7,8-DHF was measured by western blotting. (**B**) Representative image and fluorescence intensity quantification of the GlyT1 expression using immunofluorescence staining. *p < 0.05, **p < 0.01, ***p < 0.001 vs. the control group. Data are expressed as the mean ± SEM. Original images of gels/blots are available in Supplementary Fig S2.

To explore whether BDNF is involved in bupivacaine-induced GlyT1 inhibition, we further investigated the effects of 7,8-DHF (BDNF receptor agonist) on GlyT1 protein expression in astrocytes. Primary astrocytes were treated with 7,8-DHF (20 nM, 40 nM, and 80 nM) for 2 h. Treatment with 7,8-DHF decreased GlyT1 expression in astrocytes, especially in the 80 nM group (*p < 0.05, **p < 0.01, ***p < 0.01, Fig. 2A). Similarly, compared with that of the control group, immunofluorescence staining indicated that GlyT1 expression was reduced in astrocytes after treatment with 40 nM 7,8-DHF, which was consistent with the results of bupivacaine treatment (*p < 0.05, **p < 0.01, Fig. 2B).

### Bupivacaine promoted AMPK activation in astrocytes

**Figure 3:**
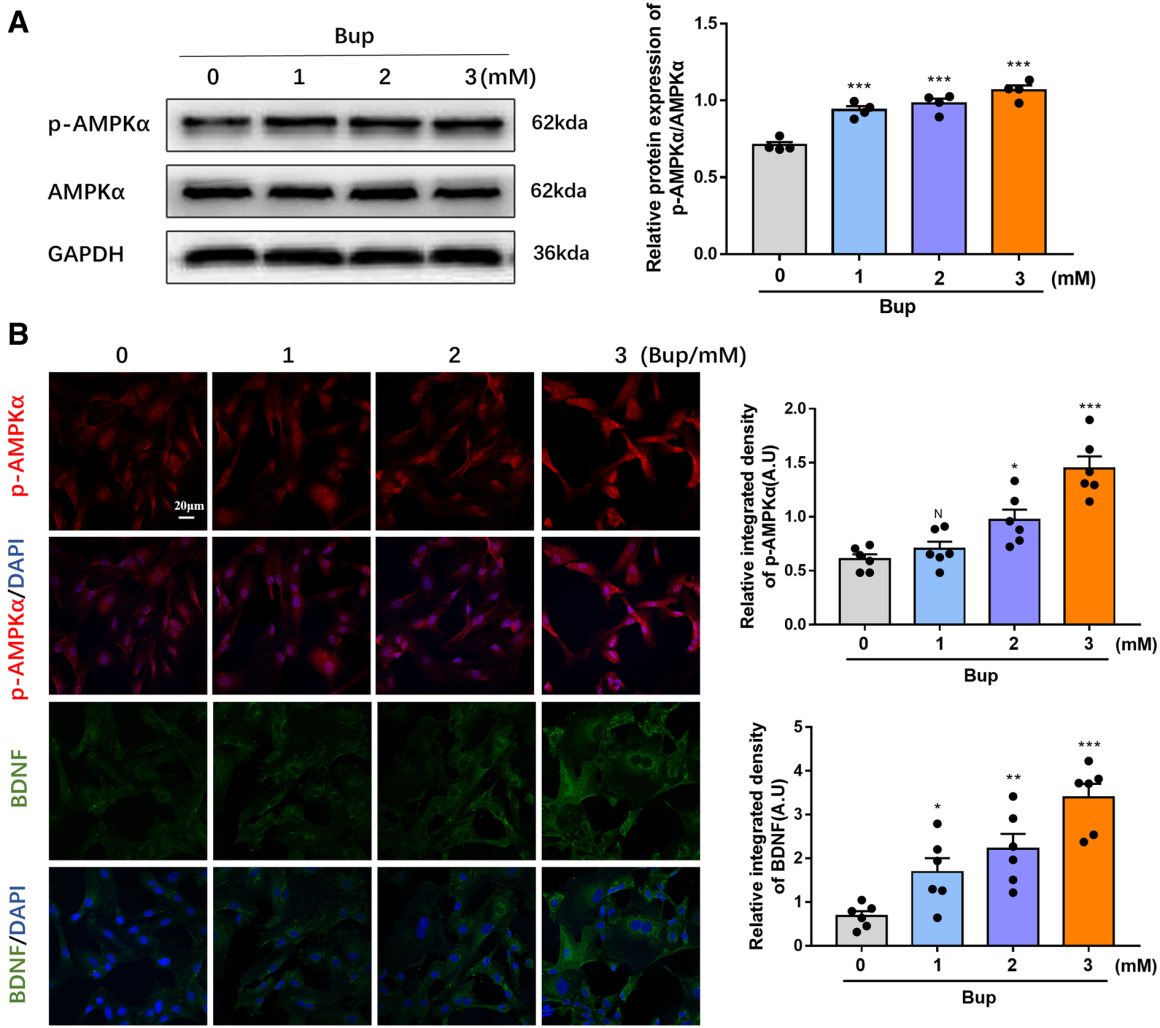
Bupivacaine activated AMPK protein in astrocytes. (**A**) Representative western blotting of p-AMPKα and AMPKα, and the ratio of p-AMPKα to total AMPK. (**B**) Immunofluorescence staining of p-AMPKα and BDNF in astrocytes after bupivacaine exposure. *p < 0.05, **p < 0.01, ***p < 0.001 vs. the control group. Data are expressed as the mean ± SEM. Original images of gels/blots are available in Supplementary Fig S3.

BDNF expression is modulated by p-AMPKα^18,21^. Furthermore, the local anaesthetic bupivacaine increased p-AMPKα expression in the CNS^24^. Therefore, we speculated that bupivacaine might activate AMPK to increase BDNF expression. Primary astrocytes were treated with bupivacaine (1 mM, 2 mM, and 3 mM) for 2 h. In line with a previous study, treatment with bupivacaine significantly increased the phosphorylation levels of AMPK, especially in the 3 mM bupivacaine group (*p < 0.05, **p < 0.01, ***p < 0.001, Figs. 1B, 3A). Similar results were obtained by immunofluorescence (*p < 0.05, **p < 0.01, ***p < 0.001, Fig. 3B).

### AMPK knockdown reversed the bupivacaine-induced enhancement of BDNF

**Figure 4:**
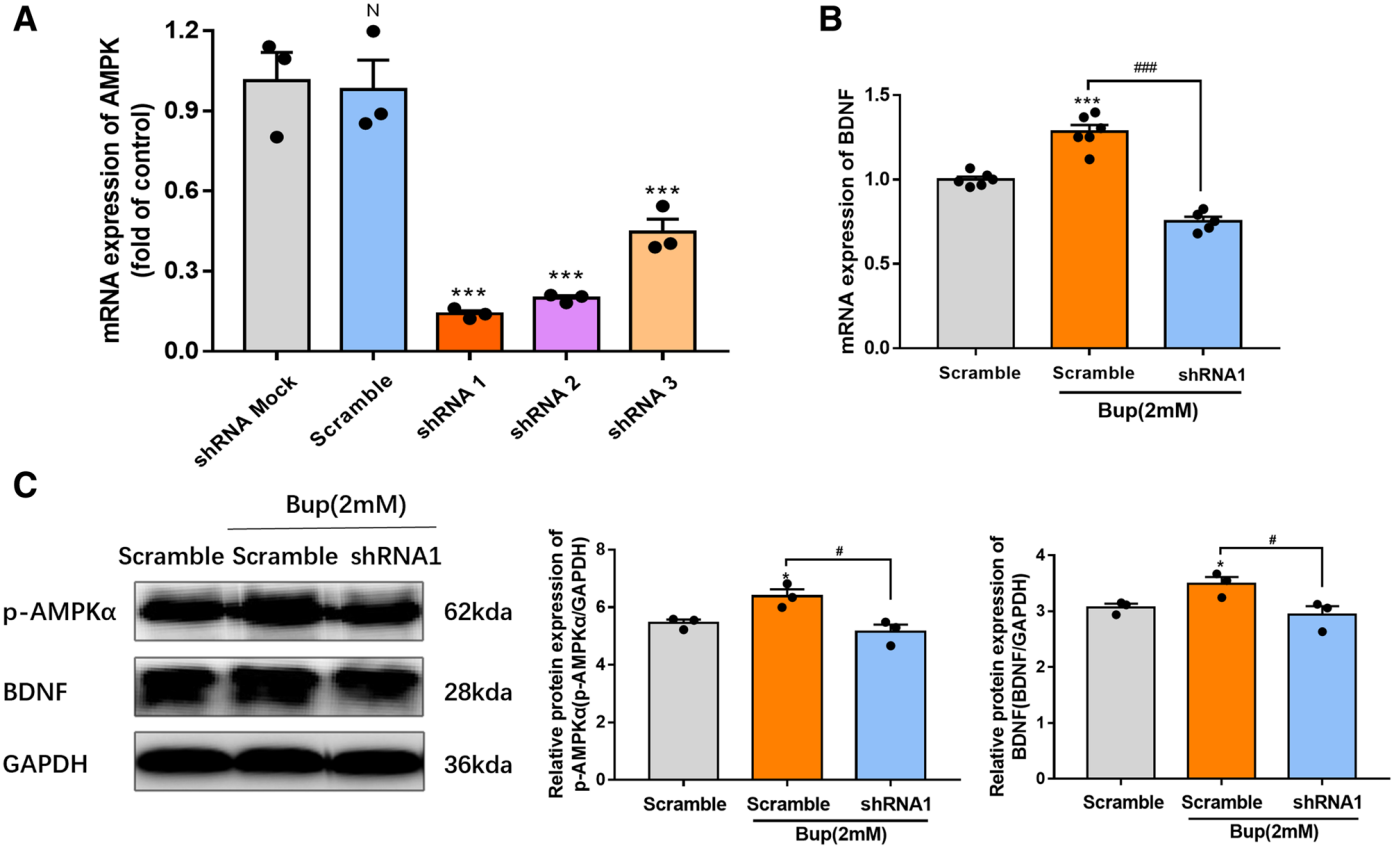
AMPK shRNA reversed the increase in BDNF by bupivacaine. (**A**) q-PCR analysis of AMPK mRNA expression in astrocytes after treatment with LV- AMPK-shRNA (shRNA1, shRNA2, shRNA3). (**B**) The expression of BDNF mRNA was measured by qPCR after bupivacaine and shRNA treatment. (**C**) Representative western blotting and relative expression of p-AMPKα and BDNF in astrocytes after bupivacaine and shRNA administration. *p < 0.05, **p < 0.01, ***p < 0.001 vs. the scram group. Data are expressed as the mean ± SEM. Original images of gels/blots are available in Supplementary Fig S4.

**Figure 5:**
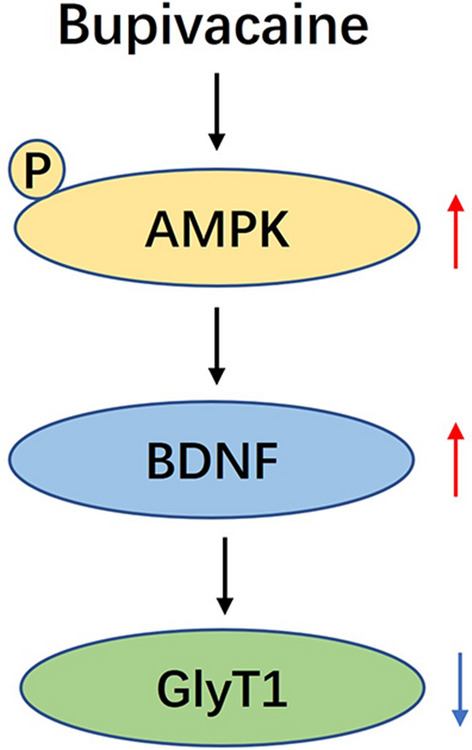
Schematic diagram of the signalling pathways involved in bupivacaine induced GlyT1 reduction in spinal astrocytes. Bupivacaine activated AMPK, and increased the expression of the downstream molecule BDNF to inhibit GlyT1 expression.

To confirm whether bupivacaine increased BDNF expression by activating AMPK, we further demonstrated the effects of AMPK-shRNA on BDNF protein expression in bupivacaine-treated astrocytes. LV- AMPK-shRNA (shRNA1, shRNA2, shRNA3) was used to knock down AMPK expression, and LV-scrambled–shRNA was used as a negative control. qPCR showed that shRNA1 had the strongest inhibition of AMPK expression (***p < 0.001) (Fig. 4A). Therefore, shRNA1 was used for AMPK knockdown. As shown in Fig. 4B, shRNA1 significantly reversed the bupivacaine-induced increase in BDNF mRNA expression (***p < 0.001, ^###^*p* < 0.001). In addition, treatment with shRNA1 reversed the bupivacaine-induced increase in BDNF protein expression (*p < 0.05, ^#^*p* < 0.05, Fig. 4C). These results indicated that bupivacaine increased BDNF expression by activating AMPK, and bupivacaine-induced enhancement of BDNF expression decreased GlyT1 expression (Fig. 5).

## Discussion

This study reported the actions of bupivacaine on GlyT1 protein expression and its further mechanism of regulation. In the CNS, GlyT1 was predominantly expressed in astrocytes, so primary astrocytes derived from the rat spinal cord were studied. Finally, we found that bupivacaine reduced GlyT1 expression in spinal astrocytes, which was associated with the potentiation of the p-AMPKα/BDNF signalling pathway.

The analgesic effects of intrathecal bupivacaine have been described but cannot be explained solely by its blockade of voltage-gated Na^+^ channels. In addition to blocking Na^+^ channels, bupivacaine can also modulate many other membrane proteins, such as NMDA receptors^26,27^, nicotinic acetylcholine and 5-HT3A receptors^28^. In addition, GlyT1-mediated glycine uptake modulated by local anaesthetic has been investigated^7,29^. However, it has not yet been established whether the antinociceptive mechanism of intrathecal bupivacaine is related to GlyT1 regulation. Therefore, the aim of the study was to investigate the effect of bupivacaine on GlyT1 in vitro to elucidate the possible mechanism of the analgesic effect of intrathecal bupivacaine.

The restoration of inhibitory glycinergic neurotransmission in the spinal cord plays a crucial role in the treatment of pathologic pain^30,31^. Glycinergic neurotransmission is controlled by GlyT1 and GlyT2. GlyT1 affects glycinergic neurotransmission by removing the glycine concentration at the glycinergic synaptic cleft, while GlyT2 mediates the reuptake of glycine into glycinergic nerve terminals. Inhibitions of GlyT1 and GlyT2 both have significant therapeutic effects on various pathologic pain conditions^32^. In particular, inhibition of GlyT1 can attenuate neuropathic pain by priming NMDA receptors for internalization^17,33,34^. In addition, it has been established that GlyT1 inhibitors can relieve pain sensitivity in different neuropathic pain models. This phenomenon is related to the enhancement of glycinergic neurotransmission and the inhibition of glutamatergic neurotransmission. A study in rats demonstrated that sciatic nerve constriction injury–induced neuropathic pain was attenuated by intrathecal GlyT1 inhibitors^35^. In addition, Armbruster et al.^36^ found that GlyT1 inhibition by intraperitoneal or oral bitopertin (GlyT1 inhibitor) could effectively alleviate hyperalgesia in rats with neuropathic pain induced by peripheral nerve injury. Furthermore, in sciatic nerve ligation injury-induced neuropathic pain models, mechanical allodynia was ameliorated by intrathecal sarcosine (GlyT1 inhibitor) and GlyT1 knockdown of the spinal cord of mice^37^. The authors of these studies concluded that inhibition of GlyT1 in the spinal cord represents a new therapy for neuropathic pain. In this study, we found that bupivacaine dose-dependently reduced GlyT1 expression in spinal astrocytes at concentrations reported in previous studies^4,5^. According to our results, a possible mechanism of the bupivacaine-mediated antinociceptive effect is the inhibition of GlyT1 expression. Nevertheless, whether bupivacaine could regulate GlyT2 has to be elucidated in further investigations.

It has been reported that many factors affect GlyT1 expression in astrocytes. Aroeira et al. reported that BDNF promoted GlyT1 internalization and degradation and suppressed GlyT1-mediated glycine uptake in astrocytes by acting on TrkB receptors^18^. Several investigations have been conducted to demonstrate that the activation of protein kinase C (PKC) induced endocytosis and degradation of GlyT1 by promoting ubiquitination of GlyT1^38^. Additionally, another study proposed that calmodulin was involved in the regulation of GlyT1 surface expression and GlyT1-mediated glycine uptake^39^. In our study, we found that bupivacaine increased the expression of BDNF and decreased GlyT1 expression in astrocytes. Moreover, GlyT1 expression in astrocytes was reduced by 7,8-DHF. Therefore, we concluded that bupivacaine reduced GlyT1 expression by upregulating BDNF expression in astrocytes. However, whether bupivacaine reduces GlyT1 expression by modulating PKC or calmodulin remains to be studied.

BDNF is one of the major neurotrophic factors produced by astrocytes to maintain the development and survival of neurons in the CNS. A previous study demonstrated that BDNF protected astrocytes from death through the TrkB signalling pathway and induced astrocytes to release neuroprotective factors^40^. In addition, BDNF plays an important role in astrocyte morphogenesis via astrocytic TrkB receptors^20,41,42^. Furthermore, many investigations have indicated that the expression of BDNF in brain astrocytes is promoted by the activation of AMPK^43,44^. AMPK is expressed ubiquitously and is a key regulator of metabolic pathways such as fatty acid and cholesterol synthesis^45^. AMPK is also active in CNS, and the activation of AMPK plays a neuroprotective or neurodegenerative role^46,47^. AMPK activation is regulated by various factors, previous studies have shown that AMPK is phosphorylated and activated by two major upstream kinases LKB1 and CAMKKβ in response to stimuli that increase intracellular AMP/ADP or calcium levels^48^. It has been reported that local anaesthetics have activating effects on AMPK^7,23^. Consistent with their findings, our study established that bupivacaine activated AMPK in spinal astrocytes, and bupivacaine increased BDNF expression by AMPK activation. But the mechanism of AMPK activation by bupivacaine remains to be further studied.

In conclusion, bupivacaine decreased GlyT1 expression in spinal astrocytes by potentiating the p-AMPKα/BDNF signalling pathway. GlyT1 inhibition of bupivacaine on spinal astrocytes provides a new molecular mechanism for the analgesic effect of intrathecal bupivacaine. However, whether the mechanism of the antinociceptive effects of intrathecal bupivacaine is consistent with our in vitro study needs to be further clarified.

## Materials and methods

### Primary cultures of astrocytes

Newborn Sprague–Dawley (SD) rats were purchased from the Laboratory Animal Center of Ningxia Medical University. All studies were approved by the Ethics Committee of Ningxia Medical University and performed according to the American Animal Protection Legislation. The study was carried out in compliance with the ARRIVE guidelines. 60 newborn rats were used in the experiment, and the age of the newborn rats was 1–2 days. Spinal cord primary astrocytes from 36 rats were used to study the effects of bupivacaine on GlyT1, BDNF and P-AMPK protein expression, and spinal cord primary astrocytes from 12 rats were applied to investigate the effect of 7,8-DHF on GlyT1 expression, spinal cord primary astrocytes from the remaining 12 mice were used to study the effect of bupivacaine on BDNF regulation after AMPK knockdown. The neonatal rats were anaesthetized by inhaling nitrous oxide. After disinfection with 75% ethyl alcohol, primary astrocytes of the spinal cord were isolated from newborn rats and cultured according to the work of Zhou et al.^49^. Briefly, the spinal cords were dissected and digested with 0.25% trypsin (Gibco) for 6 min at 37 °C, the reaction was terminated by adding medium containing foetal bovine serum, and the cell suspension was centrifuged at 1200 rpm for 5 min. Finally, the cell pellet was cultured in a mixed medium of DMEM/F12 (1:1) with 10% FBS and 1% penicillin/streptomycin, and the cells were cultured in 75 cm^2^ polylysine-coated flasks in the presence of 5% CO_2_. Nonastrocytes were detached from the flasks by shaking and were removed by changing the medium. Third- or fourth-passage cells were rendered quiescent through incubation in medium containing 0.5% FBS for 4 days prior to the experiments. Astrocytes were confirmed by their typical morphology and positive staining for the specific marker GFAP, and the purity of astrocytes was over 90% (see Supplementary Fig S5).

### Drugs and treatments

Bupivacaine hydrochloride was provided by Zhaohui Pharmaceutical (Shanghai, China). Treatment with 7,8-dihydroxyflavone (7,8-DHF), a specific TrkB receptor (one of the BDNF receptors) agonists, mimicked the physiological effects of BDNF. It was purchased from MedChemExpress. Bupivacaine was diluted to 1 mM, 2 mM, and 3 mM with DMEM/F12 medium containing 0.5% FBS and then treated with primary astrocytes for 2 h. The concentration of bupivacaine was determined according to the research of Huang et al. ^24^. Moreover, 7,8-DHF was diluted to 5 ng/mL, 10 ng/mL, and 20 ng/mL with DMEM/F12 medium containing 0.5% FBS and administered to primary astrocytes for 2 h. The concentration of 7,8-DHF was determined by CCK-8 assays for cell viability (see Supplementary Fig S6). Then, various experimental tests were carried out.

### Cell transfection with shRNA

For knockdown of AMPK, the primary astrocytes were transfected with lentivirus vector containing AMPK short hairpin RNA and a green fluorescent protein expression marker (LV- AMPK-shRNA–green fluorescent protein; 5 × 10^8^ TU/mL) to knock down AMPK expression and transfected with lentivirus expression vector containing scrambled–short hairpin RNA–green fluorescent protein (LV-scrambled–shRNA–green fluorescent protein; 5 × 10^8^ TU/mL) as a negative control. The nucleotide sequences of short hairpin RNAs (shRNAs) targeting AMPK were as follows: 5'-GCCGACTTCGGTCTTTCAAAC-3' (shRNA1), 5'-GGACTATGAATGGAAGGTTG T-3' (shRNA2), and 5'-GCAACAAGCCCACCCGATTCT-3' (shRNA3). A scrambled sequence was also designed as a negative control (Scram): 5'-TTCTCCGAA CGTGT CACGT-3' (Scram). The lentivirus vectors were designed and generated by Genomeditech (Shanghai, China).

Primary astrocytes were diluted to a density of 2 × 10^4^ cells/ml, seeded in new plates and incubated until 70–80% confluent cells were reached. The viral vector containing shRNA was diluted to the corresponding concentration according to MOI = 50 and incubated with primary astrocytes for 48 h. Then, follow-up experiments were performed after 48 h of cell recovery. This method was used to knock down AMPK expression in primary astrocytes.

### Quantitative real-time PCR

Total RNA was extracted from astrocyte cultures by using a Total RNA Mini Isolation Kit (Axygen; Corning Incorporated, USA) according to the manufacturer’s protocol. The cDNA templates were synthesized by the One-Step gDNA Removal kit (TransGen Biotech, China) according to the manufacturer’s instructions. All real-time PCRs were performed using TransStart TipTop Green qPCR SuperMix (TransGen Biotech, China) and carried out by a Jena quantitative PCR instrument (qTOWER2.0, Jena, Germany). The list of specific primers applied for amplifying genes is shown in Supplementary Table S1. The relative gene expression values were calculated using the comparative 2^-ΔΔCq^ method, and GAPDH was used as a control.

### Western blotting analysis

Primary cultured astrocytes were lysed in lysis buffer (KeyGEN Biotech, Nanjing, China) containing protease and phosphatase inhibitors for 30 min. After centrifugation, the supernatant was collected for protein quantification by the BCA protein assay (KeyGEN Biotech, Nanjing, China). The protein extracts with sodium dodecyl sulphate (SDS) sample buffer were heated in a 100 °C water bath for 10 min. The samples (40 μg sample^-1^) were loaded on 10% SDS-PAGE for electrophoresis, and then, the gel that contained the proteins was cut according to the protein molecular size. Next, the same proteins come from two pieces of gel were transferred onto a PVDF membrane. The membrane was blocked using 5% bovine serum albumin (BSA) in Tris-buffered saline with Tween (TBS-T; 10 mmol litre^-1^ Tris–HCl, 150 mmol litre-^1^ NaCl, 0.1% Tween; pH 8.0) for 2 h. Next, the membrane was probed overnight at 4 °C with primary antibodies including rabbit anti-GlyT1 (1:500; Proteintech Group, Inc.), rabbit anti-BDNF (1:500; Proteintech Group, Inc.), rabbit anti-AMPKα (1:500; Proteintech Group, Inc.), and rabbit anti-p-AMPKα (Thr172) (1:500; Affinity Biosciences, Inc.), followed by HRP-conjugated goat anti-rabbit IgG (1:3000; Proteintech Group, Inc.) for 2 h. Blots were visualized with an enhanced chemiluminescence (ECL) system (Pierce; Thermo Fisher Scientific, Inc.), and the protein bands were quantified by Quantity One software version 4.6.2. Normalization was performed with GAPDH expression.

### Immunofluorescence staining and microscopy

For immunocytochemistry assays, primary astrocytes were fixed with 4% paraformaldehyde (PFA) for 15 min at room temperature. All the cells were blocked with 5% goat serum in 0.01 mM phosphate-buffered saline (PBS, pH 7.4) with 0.3% Triton-X-100 for 2 g h at room temperature and then incubated overnight at 4 °C with primary antibodies, including mouse anti-GlyT1 (1:25; Proteintech Group, Inc.), mouse anti-BDNF (1:50; Proteintech Group, Inc.), and rabbit anti-p-AMPKα (Thr172) (1:50; Affinity Biosciences, Inc.). Then, the cells were incubated with TRITC–conjugated goat anti-rabbit IgG or FITC–conjugated goat anti-mouse IgG (1:100; Proteintech Group, Inc.) for 2 h at room temperature. Finally, the cells were sealed with an antifluorescence attenuating tablet containing DAPI. Omission of the primary antibody served as a negative control. Images were captured by confocal laser scanning microscopy (FluoView FV 1000; Olympus, Tokyo, Japan). Image-Pro Plus 6.0 software was applied to quantify the fluorescence intensity of images, and GraphPad Prism 7.0 software was used to generate graphs.

### Statistical analysis

Data were analysed using SPSS 22.0 statistical software. All data are expressed as the mean ± SEM. All experiments were repeated at least three times. One-way ANOVA was used to compare the means of multiple samples. Statistical significance of the results was defined at *p* < 0.05.

### Ethics approval and consent to participate

The protocol was approved by the Ethics Committee of Ningxia Medical University (date from 2021.6.1 to 2023.5.31, protocol number 2020–834). All experimental processes complied with internationally accredited guidelines and ethical regulations.

## Supplementary Information

Supplementary Information.

## Data Availability

The datasets used and analysed during the current study are available from the corresponding author on reasonable request.
