# Supplementary material for: Bupivacaine reduces GlyT1 expression by potentiating the p-AMPKα/BDNF signalling pathway in spinal astrocytes of rats
Source: Sci Rep. 2022 Jan 26;12:1378. doi: 10.1038/s41598-022-05478-3 (PMC8792009; doi:10.1038/s41598-022-05478-3)
Supplement: Supplementary file 1 — Supplementary Information. [file 41598_2022_5478_MOESM1_ESM.pdf]

Bupivacaine Reduces GlyT1 Expression by Potentiating the p-AMPKα/ BDNF Signaling Pathway in Spinal Astrocytes of Rats

Kaimei Lu[1], Liyan Zhao[2], Yonghai Zhang[3], Fan Yang[3], Huiwen Zhang[3], Jie Wang[1], Bin Li[1], Guimei Ji[1], Jianqiang Yu[4*], Hanxiang Ma[3*]

List of Supplementary Materials

Original gels/blots of cropped images

Fig S1. Original gels/blots for Figure 1A, B.
Fig S2. Original gels/blots for Figure 2A.
Fig S3. Original gels/blots for Figure 3A.
Fig S4. Original gels/blots for Supplementary Figure 4C

Supplementary Figure.

Fig S5. Immunofluorescence staining of GFAP was used to confirm the purity of primary astrocytes.
Fig S6. Effect of 7,8-DHF on the survival rate of primary astrocytes.

Supplementary Table.

Table S1. The list of q-PCR primers used in this study

Supplementary Information

Fig S1. Original gels/blots for Figure 1A, B.

A

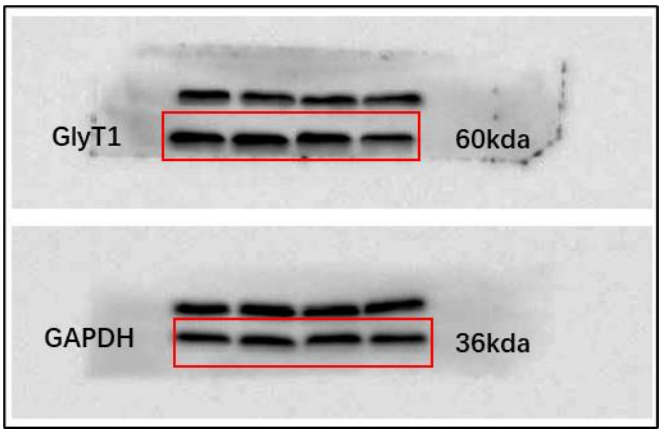

B

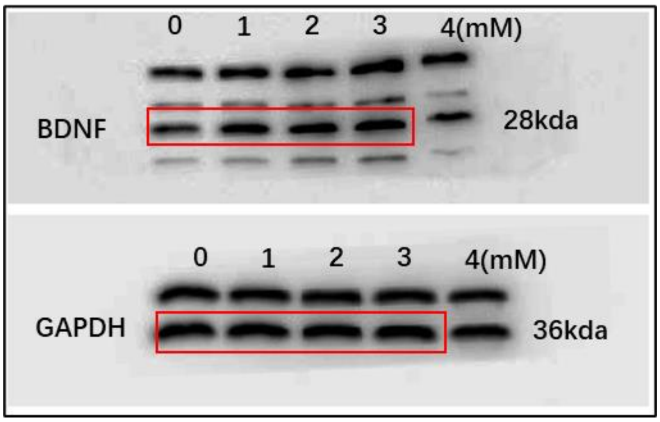

Fig S2. Original gels/blots for Figure 2A.

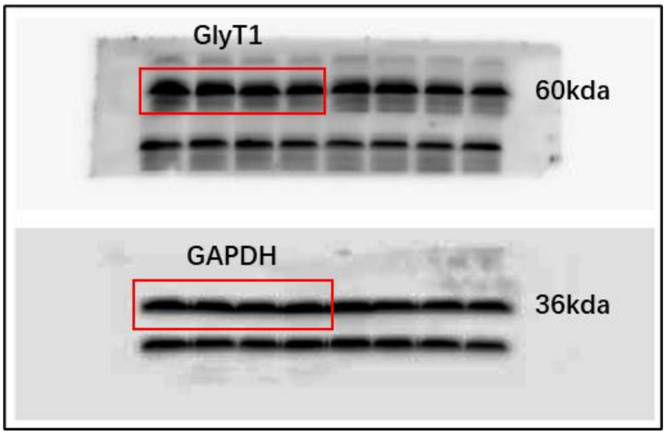

Fig S3. Original gels/blots for Figure 3A

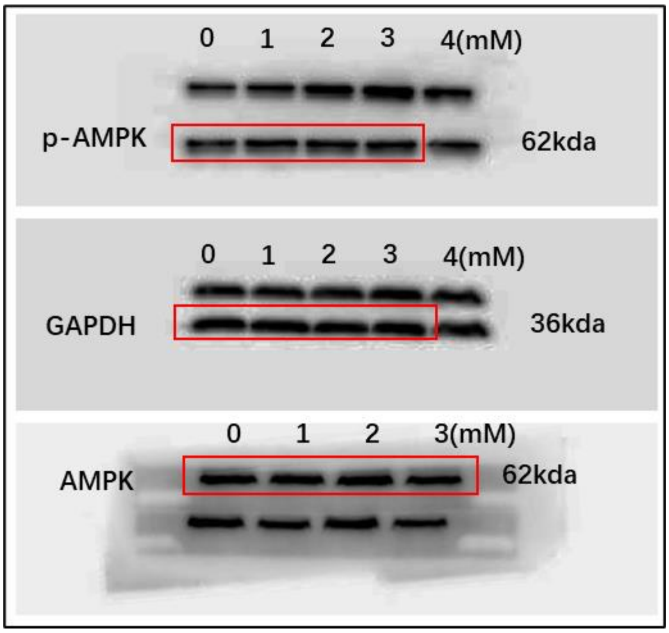

Fig S4. Original gels/blots for Figure 4C.

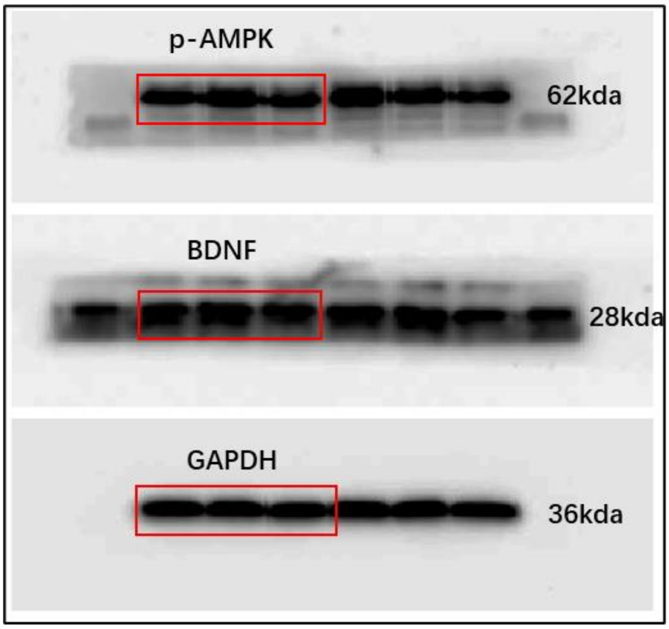

Fig S5. Immunofluorescence staining of GFAP for primary cultures, the purity of astrocytes was determined by calculating the ratio of red cells to total cells. Finally, the purity of astrocytes was over 90%, then the astrocytes were used to the following experiments.

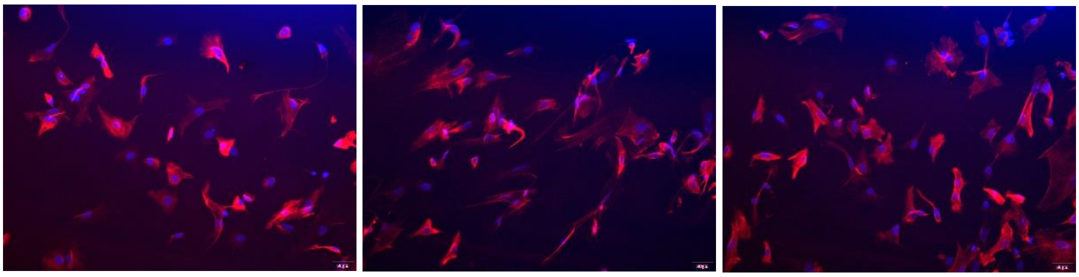

Fig S6. Effect of 7,8-DHF on the survival rate of primary astrocytes. cck8 analysis was used to detect the viability of primary astrocytes under treatment with different dose of 7,8-DHF for 2 h (n=6, per group). Finally, we choesed 20, 40, 80 nM 7,8-DHF for the study.

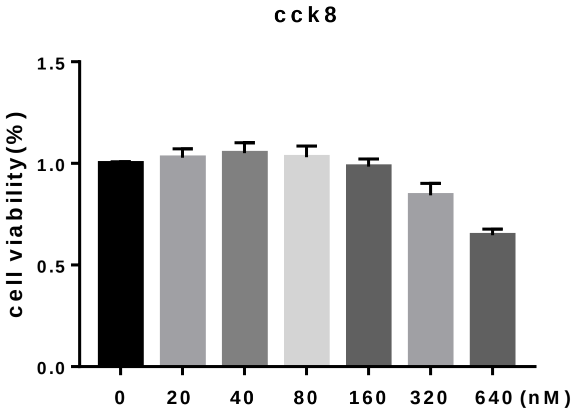

Table S1. Primer sequences and annealing temperatures.

Primer	Forward sequence (5'-3')	Reverse sequence (5'-3')	Annealing temperature, ˚C
BDNF	TTCTACGAGACCAAGTATAATCC	TTATGAACCGCCAGCCAATT	59
AMPK	CCCGACACACCCTAGATGAATTA AACC	TAGTCCAACTGCTTGATTGCT CTACAC	59
GAPDH	ACAGCAACAGGGTGGTGGAC	TTTGAGGGTGACGCGAACTT	59

BDNF, brain-derived neurotrophic factor; AMPK, adenosine 5′-monophosphate (AMP)-activated protein kinase;
